# Assessment of geraniol-incorporated polymers to control *Aedes albopictus* (Diptera: culicidae)

**DOI:** 10.1051/parasite/2012194427

**Published:** 2012-11-15

**Authors:** T. Chuaycharoensuk, S. Manguin, G. Duvallet, T. Chareonviriyaphap

**Affiliations:** 1 Department of Entomology, Faculty of Agriculture, Kasetsart University Bangkok 10900 Thailand; 2 Institute of Research for Development (IRD), Laboratory of Molecular Immuno-physiology (UMR-MD3), University Montpellier1-2 Montpellier France; 3 Centre for Functional and Evolutionary Ecology (UMR 5175- CEFE), Paul-Valery University Montpellier France

**Keywords:** *Aedes albopictus*, mosquito vector, larvicide, monoterpene, natural product, vector control, *Aedes albopictus*, vecteur, larvicide, monoterpène, produit naturel, lutte antivectorielle

## Abstract

Effective control of mosquito borne diseases has proven extremely difficult with both vector and pathogen remaining entrenched and expanding in many disease endemic areas. When lacking an effective vaccine, vector control methods targeting both larval habitats and adult mosquito populations remain the primary strategy for reducing risk. *Aedes albopictus* from Thailand was used as a reference baseline for evaluation of natural insecticides incorporated in polymer disks and pellets and tested both in laboratory and field conditions. In laboratory and field tests, the highest larval mortality was obtained with disks or pellets containing IKHC (Insect Killer Highly Concentrate) from Fulltec AG Company. This product is reputed to contain geraniol as an active ingredient. With pellets, high mortality of *Ae. albopictus* larvae (92%) was observed in presence of 1 g of pellets per 500 ml of water at day 1st, and the mortality was 100% at day 1st for larvae in presence of 5 or 10 g of pellets. Fulltec AG Company has not accepted to give us the exact composition of their IKHC product. Therefore, we cannot recommend it, but the principle of using monoterpenes like geraniol, incorporated into polymer disks or pellets as natural larvicide needs more attention as it could be considered as a powerful alternative in mosquito vector control.

## Abstract (fr)

Le contrôle effectif des maladies à transmission vectorielle est très difficile, les vecteurs et les pathogènes se maintenant souvent en position retranchée et se développant dans de nombreux foyers endémiques. En l’absence de vaccin efficace, les méthodes de lutte antivectorielle ciblant à la fois les habitats larvaires et les populations d’adultes restent la stratégie de choix pour réduire le risque. *Aedes albopictus* en Thaïlande a été choisi pour des tests d’évaluation d’insecticides naturels incorporés dans des disques ou des granulés de polymères, à la fois au laboratoire et sur le terrain. Dans tous les tests, la mortalité larvaire la plus élevée a été obtenue avec les disques et les granulés contenant de l’IKHC (*Insect Killer Highly Concentrate*) de la compagnie Fulltec AG. Ce produit est réputé contenir du géraniol comme matière active. Avec les granulés, une mortalité de 92% a été observée en présence d’un gramme de granulés pour 500 ml d’eau dès le premier jour, et cette mortalité était de 100% en présence de 5 et 10 g de granulés dès le premier jour. La compagnie Fulltec AG n’ayant pas accepté de nous donner la composition exacte de leur IKHC, nous ne pouvons recommander ce produit. Mais le principe d’utiliser des monoterpènes incorporés dans des disques ou des granulés de polymères, comme traitement larvicide, nécessite une grande attention, car cela pourrait devenir une méthode alternative efficace de contrôle des moustiques.

Since the implementation of the European directive 98/8/EC, known as the “biocide directive”, most of the old effective, inexpensive but polluting biocides used for vector control (Rozendaal, 1997) have been withdrawn from the European market. Now, assessment of unwanted side-effects is compulsory for any active biocide substance and, eventually, for any formulated biocide product that contains it, prior to acquiring marketing authorization. Considerations of cost, acceptability, safety and, more recently, respect for the environment, have led companies, research institutes and international agencies to offer new compounds such as insecticides of biological origin, like *Bacillus thuringiensis* ser. *israelensis* (Bti), *Bacillus sphaericus*, Spinosad and growth regulators mimicking insect hormones (Fontenille *et al.*, 2009). Certain active pyrethroid substances show the advantage of being active at low doses and having low persistence (no residual effect), but have low selectivity. Some trials are also under way on natural substances such as essential oils or plant extracts, like geraniol (Khallaayoune *et al.*, 2009; George *et al.*, 2009) or other monoterpenes. Most compounds used for vector control are implemented by trained specialized teams, but after the epidemics of chikungunya in La Reunion Island (2005-2006) and dengue in Martinique and Guadeloupe, personal protection against vectors (PPAV) revealed to be also very important (PPAV Working Group, 2011). The main dengue and chikungunya vectors like *Aedes aegypti*, *Ae. albopictus* (Guzman *et al.*, 2010) and also nuisance caused by *Culex quinquefasciatus*, could be partly controlled by mechanical elimination of breeding sites around houses and in gardens, and/or by larvicide treatment and spatial spraying with insecticides. To implement a larvicide treatment by people, some companies are developing monoterpenes-incorporated polymer disks or pellets to be used in gardens or terraces. The efficiency of some of these products has been assessed in Thailand at the Entomology Research Laboratory of Kasetsart University, Bangkok, in laboratory conditions where mosquito colonies are maintained, as well as in field conditions where *Ae. albopictus* is prevalent.

## Materials

### *Aedes Albopictus* Colony

*Ae. albopictus* MOPH colony (Thailand) was used as a reference baseline for these tests. This colony was originally collected from Nonthaburi Province and has been maintained in a colony at Vector Borne Disease Bureau, Disease Control Deparment, Ministry of Public Health (MOPH, Thailand) for approximately ten years (Chuaycharoensuk *et al.*, 2011). Mosquito colony was carefully segregated to prevent contamination from cross-mating and reared under identical laboratory-controlled condition at 25 ± 5 °C and relative humidity 80 ± 10 % in the Department of Entomology, Faculty of Agriculture, Kasetsart University, Bangkok, Thailand (Kongmee *et al.*, 2004). Adult mosquitoes were allowed to mate and females permitted to blood feed on restrained live guinea-pigs beginning on the fourth day postemergence. Moistened oviposition substrates were provided to the females for depositing their eggs. Following conditioning, eggs were re-hydrated and allowed to hatch in pans reserved for each strain. In the present experiment, only stage 3 mosquito larvae were available in sufficient numbers at the time of the test and used.

### Geraniol Incorporated Disks and Pellets

Polymer disks and pellets were sent to us under sealed aluminium bags by Valcoplast SA (4, Avenue Bordaberri, 64990 Mouguerre, France) without any indication on their composition for evaluation in blind conditions. The composition was revealed after the tests were done. The disks were made of polymer with incorporation of different products (Table I). IKHC was presented by Fulltec AG (Zug, Switzerland) as a watery concentrate with geraniol (CAS 106-24-1) from natural origin as an active ingredient. Geraniol is extracted by chromatography from palmarosa essential oil (Java Lemongrass, *Cymbopogon martinii*). The final concentration of the products incorporated in the polymer disks was around 10 %. The pellets of 0.5 cm of diameter were made of a mixture of polymer and wood fibers impregnated with IKHC.

**Table I.** Details on the products incorporated into the disks.

		Fulltec AG Company, Switzerland		Penta Manufacturing Company, USA
Disks		IKHC*	Synthetic geraniol	Synthetic geraniol
1903/1	Control			
1903/2			100 %	
1903/3				100 %
1903/4		95 %	5 %	
1903/5		95 %		5 %
1903/6		50 %	50 %	
“Juillet2010”		100 %		

* Insect Killer Highly Concentrate G.

## Methods

### In The Laboratory

Stage 3 mosquito larvae were tested in plastic saucers. In the Experiment A, 50 larvae, 500 ml of filtered water, two tablets of fish food and one polymer disk were added in each saucer. In this experiment, three replicates were done for each of the polymer disk lot. The number of dead larvae was counted every day during one week (day 6 for practical reasons). Afterwards, the polymer disks were kept dry at room temperature and experiment was repeated after one and two months to evaluate the remanence of the effect. A similar experiment (Experiment B) was performed at the same time with polymer disks replaced by IKHC incorporated pellets. One, two or five grams of pellets were added to each saucer containing 500 ml of filtered water. As in Experiment A, 50 stage 3 larvae and two tablets of fish food were added in each saucer. A saucer without pellets was incorporated as a control. Two replicates were done. The number of dead larvae was counted every day until all larvae were dead in all saucers with pellets.

### In The Field

In this experiment saucers were placed in the garden near the laboratory and protected from rain (Experiment C). Wild populations of *Ae. albopictus* are abundant in this area. The saucers were filled in with 500 ml of filtered water and two tablets of fish food. There were two replicates for each polymer disk lot. The presence of new emerged larvae was checked until Day 18 (D18). This experiment was also repeated after one and two months like Experiment A.

## Results

For Experiments A and B, results are expressed as larval reduction rates of stage 3 *Ae. albopictus* for each of the polymer disk or pellet lots, with indication of standard deviation. For Experiment C, results are expressed as presence and abundance of larvae after exposure of the saucers in the garden.

In Experiment A (Table II), a very low larval mortality was noticed in lot 1903/1 (control) until D6. A very low mortality was also observed for lots 1903/2 and /3 with 2.7 % and 4.7 % respectively at D6. On the opposite, a strong mortality was observed with lots 1903/4, /5 and “Juillet2010” with 99 %, 94 % and 100 % respectively at D3 and 100 % afterwards. For lot 1903/6, the mortality was low until D3 and increased dramatically onwards to 50 % at D4 and 91 % at D6.

**Table II.** Experiment A: larval reduction rates (%) of *Ae. albopictus* in presence of geraniol impregnated polymer disks.

		Different polymer disks						
	Dates	1903/1*	1903/2	1903/3	1903/4	1903/5	1903/6	“Juillet2010”
D0	05/04/2011	0 ± 0**	0 ± 0	0 ± 0	0 ± 0	0 ± 0	0 ± 0	0 ± 0
D1	06/04/2011	0.7 ± 1.2	0 ± 0	0 ± 0	83 ± 29	70 ± 20	0 ± 0	97 ± 5.8
D2	07/04/2011	2 ± 2	0 ± 0	2.7 ± 4.6	96 ± 6.9	89 ± 9.9	4.7 ± 4.6	96 ± 4
D3	08/04/2011	2 ± 2	0.7 ± 1.2	2.7 ± 4.6	99 ± 1.2	94 ± 8.7	4.7 ± 4.6	100 ± 0
D4	09/04/2011	2 ± 2	0.7 ± 1.2	2.7 ± 4.6	100 ± 0	100 ± 0	50 ± 38	100 ± 0
D5	10/04/2011	ND	ND	ND	ND	ND	ND	ND
D6	11/04/2011	2 ± 2	2.7 ± 2.3	4.7 ± 5	100 ± 0	100 ± 0	91 ± 12	100 ± 0

* See text for the identification of polymer disks.

** All figures are means of triplicates ± SD. ND: not done.

**Table II bis:** Experiment A: larval reduction rates (%) of *Ae. albopictus* with the same geraniol impregnated polymer disks. 1 month after the previous test.

		Different polymer disks						
	Dates	1903/1*	1903/2	1903/3	1903/4	1903/5	1903/6	“Juillet2010”
D0	07/05/2011	0 ± 0	0 ± 0	0 ± 0	0 ± 0	0 ± 0	0 ± 0	0 ± 0
D1	08/05/2011	0 ± 0	0.7 ± 1.2	0.7 ± 1.2	2 ± 3.5	3.3 ± 5.8	0 ± 0	8.7 ± 1.2
D2	09/05/2011	0 ± 0	0.7 ± 1.2	1.3 ± 1.2	4.7 ± 3.1	6 ± 5.3	2 ± 2	11 ± 3.1
D3	10/05/2011	0 ± 0	0.7 ± 1.2	1.3 ± 1.2	5.3 ± 2.3	8.7 ± 3.1	2.7 ± 1.2	13 ± 3.1
D4	11/05/2011	0.7 ± 1.2	0.7 ± 1.2	1.3 ± 1.2	8.7 ± 3.1	9.3 ± 2.3	4 ± 2	15 ± 1.2
D5	12/05/2011	0.7 ± 1.2	0.7 ± 1.2	1.3 ± 1.2	8.7 ± 3.1	11 ± 3.1	4 ± 2	18 ± 3.5
D6	13/05/2011	0.7 ± 1.2	0.7 ± 1.2	2 ± 0	11 ± 3.1	14 ± 2	4.7 ± 1.2	19 ± 4.2

**Table II ter:** Experiment A: Larval reduction rates (%) of *Ae. albopictus* with the same geraniol impregnated polymer disks. two months after the first test.

		Different polymer disks						
	Dates	1903/1	1903/2	1903/3	1903/4	1903/5	1903/6	“Juillet2010”
D0	05/06/2011	0 ± 0	0 ± 0	0 ± 0	0 ± 0	0	0 ± 0	0 ± 0
D1	06/06/2011	0 ± 0	0 ± 0	0 ± 0	2 ± 3.5	2 ± 3.5	0 ± 0	8.7 ± 1.2
D2	07/06/2011	0 ± 0	0 ± 0	0 ± 0	4.7 ± 3.1	4.7 ± 3.1	2 ± 2	11 ± 3.1
D3	08/06/2011	0 ± 0	0.7 ± 1.2	0 ± 0	5.3 ± 2.3	8.7 ± 3.1	2.7 ± 1.2	13 ± 3.1
D4	09/06/2011	0.7 ± 1.2	0.7 ± 1.2	1.3 ± 1.2	8.7 ± 3.1	11 ± 3.1	6.7 ± 1.2	15 ± 1.2
D5	10/06/2011	0.7 ± 1.2	0.7 ± 1.2	2.7 ± 1.2	11 ± 3.1	14 ± 2	9.3 ± 1.2	18 ± 3.5
D6	11/06/2011	0.7 ± 1.2	1.3 ± 1.2	2.7 ± 1.2	14 ± 2	22 ± 2	13 ± 3.1	19 ± 4.2

The results of the same experiment done one month after the previous test, using the same polymer disks that were washed and kept dry at room temperature (Table II bis) showed a very low mortality (below 5 %) in lots 1903/1 (control), /2, /3 and /6; and a low mortality of 11 %, 14 % and 19 % for lots 1903/4, /5 and “Juillet2010” respectively at D6. The same experiment done two months later gave similar results (Table II ter) with a very low mortality (below 3 %) in lots 1903/1 (control), /2, /3; and a low mortality of 14 %, 22 %, 13 %, and 19 % for lots 1903/4, /5, /6 and “Juillet2010” respectively at D6.

In Experiment B (Table III), there was a very low mortality (2 % at D3) in the control saucer (without any pellet), but a very high mortality in presence of pellets. With 1 g of pellets in the saucers, the larval mortality was 92 % at D1, 98 % at D2 and 100 % at D3. With 5 and 10 g of pellets, the mortality was 100 % as soon as D1. One and two months later (Tables III bis, III ter), the mortality stays quite high with 84 % and 83 % at D1 for 1g pellets, and 100 % at D1 for both 5 g and 10 g pellets.

**Table III.** Experiment B: larval reduction rates (%) of *Ae. albopictus* in presence of geraniol impregnated pellets.

		Different mass of geraniol impregnated pellets			
	Dates	Control	1 g	5 g	10 g
D0	05/04/2011	0 ± 0*	0 ± 0	0 ± 0	0 ± 0
D1	06/04/2011	0 ± 0	92 ± 1.4	100 ± 0	100 ± 0
D2	07/04/2011	0 ± 0	98 ± 1.4	100 ± 0	100 ± 0
D3	08/04/2011	2 ± 0	100 ± 0	100 ± 0	100 ± 0

* All figures are means of duplicates (± SD).

**Table III bis:** Experiment B: larval reduction rates (%) of *Ae. albopictus* in presence of geraniol impregnated pellets, one month after previous test.

		Different mass of geraniol impregnated pellets			
	Dates	Control	1 g	5 g	10 g
D0	07/05/2011	0 ± 0	0 ± 0	0 ± 0	0 ± 0
D1	08/05/2011	0 ± 0	84 ± 1.4	100 ± 0	100 ± 0
D2	09/05/2011	0 ± 0	93 ± 0.7	100 ± 0	100 ± 0
D3	10/05/2011	0 ± 0	100 ± 0	100 ± 0	100 ± 0

**Table III ter:** Experiment B: larval reduction rates (%) of *Ae. albopictus* in presence of geraniol impregnated pellets, two months after first test.

		Different mass of geraniol impregnated pellets			
	Dates	Control	1 g	5 g	10 g
D0	07/05/2011	0 ± 0	0 ± 0	0 ± 0	0 ± 0
D1	08/05/2011	0 ± 0	83 ± 4.2	100 ± 0	100 ± 0
D2	09/05/2011	0 ± 0	89 ± 1.4	100 ± 0	100 ± 0
D3	10/05/2011	0 ± 0	96 ± 2.8	100 ± 0	100 ± 0
D4	09/04/2011	0 ± 0	100 ± 0	100 ± 0	100 ± 0

In Experiment C (Table IV), young larvae of *Ae. albopictus* were found in the control saucer (with polymer disk 1903/1) at D6 and their number increased onward. The same was found for lots 1903/2 and /3, for which larvae were found as early as D9. For lot 1903/6, larvae were found at D15 onward, but no larva was found in lots 1903/4, /5 and “Juillet2010”. One month later (Table IV bis), young larvae of *Ae. albopictus* were found in the control saucer (with polymer disk 1903/1) at D15 and increased, as well as in saucers of lots 1903/3 and /6. Larvae were found earlier for lot1903/2 at D12 and later for lot 1903/4 at D18. And no larva was found in lots 1903/5 and “Juillet2010”. Two months later (Table IV ter), young larvae of *Ae. albopictus* were found in the control saucer (with polymer disk 1903/1) and 1903/2 at D9. For lots 1903/3 and /6 larvae were found at D12, for lot 1903/4 at D15, and for lot 1903/5 at D18. No larva was found in lot “Juillet2010”.

**Table IV.** Experiment C: presence and abundance of *Ae. albopictus* larvae in saucers with different geraniol impregnated polymer disks in an infested area.

		Different polymer disks												
			1903/2		1903/3		1903/4		1903/5		1903/6		“Juillet2010”	
	Dates	1903/1	a*	b*	a	b	a	b	a	b	a	b	a	b
D0	05/04/2011	0	0	0	0	0	0	0	0	0	0	0	0	0
D3	08/04/2011	0	0	0	0	0	0	0	0	0	0	0	0	0
D6	11/04/2011	+++	0	0	0	0	0	0	0	0	0	0	0	0
D9	14/04/2011	++++	0	+++	0	+	0	0	0	0	0	0	0	0
D12	17/04/2011	+++++	0	++++	0	++	0	0	0	0	0	0	0	0
D15	20/04/2011	++++++	0	++++	++	+++	0	0	0	0	++	++	0	0
D18	23/04/2011	++++++	++	++++	+++	+++	0	0	0	0	+++	+++	0	0

* a and b are duplicates with similar polymer disks. +: 1–5 larvae; ++: 6–10 larvae; +++: 11–15 larvae; ++++: 16–20 larvae; +++++: 21–25 larvae; ++++++: 26–30 larvae.

**Table IV bis:** Experiment C: presence and abundance of *Ae. albopictus* larvae in saucers with different geraniol impregnated polymer disks in an infested area, one month after the previous test using the same disks.

		Different polymer disks												
			1903/2		1903/3		1903/4		1903/5		1903/6		“Juillet2010”	
	Dates	1903/1	a	b	a	b	a	b	a	b	a	b	a	b
D0	07/05/2011	0	0	0	0	0	0	0	0	0	0	0	0	0
D3	10/05/2011	0	0	0	0	0	0	0	0	0	0	0	0	0
D6	13/05/2011	0	0	0	0	0	0	0	0	0	0	0	0	0
D9	16/05/2011	0	0	0	0	0	0	0	0	0	0	0	0	0
D12	19/05/2011	0	0	++++	0	0	0	0	0	0	0	0	0	0
D15	22/05/2011	+++	++	+++++	++	+++	0	0	0	0	++++	+++	0	0
D18	25/05/2011	++++++	+++	++++++	++++	++++	++	0	0	0	+++++	++++	0	0

**Table IV ter:** Experiment C: presence and abundance of *Ae. albopictus* larvae in saucers with different geraniol impregnated polymer disks in an infested area, two months after the previous test using the same disks.

		Different polymer disks												
			1903/2		1903/3		1903/4		1903/5		1903/6		“Juillet2010”	
	Dates	1903/1	a	b	a	b	a	b	a	b	a	b	a	b
D0	05/06/2011	0	0	0	0	0	0	0	0	0	0	0	0	0
D3	08/06/2011	0	0	0	0	0	0	0	0	0	0	0	0	0
D6	11/06/2011	0	0	0	0	0	0	0	0	0	0	0	0	0
D9	14/06/2011	+	0	+	0	0	0	0	0	0	0	0	0	0
D12	17/06/2011	++	+	+++	0	++	0	0	0	0	++	+	0	0
D15	20/06/2011	++++	+++	++++	++	+++	+	0	0	0	++++	+++	0	0
D18	23/06/2011	++++++	+++++	+++++	++++	+++++	++	++	+	+	++++++	+++++	0	0

## Discussion and Conclusion

Polymer disks or pellets, impregnated with different geraniol based products, were tested in laboratory and in open field conditions. The main objective of this study was to check if those products could be used on an individual basis to control *Ae. albopictus* or other anthropophilic mosquito populations in gardens or terraces.

The *Ae. albopictus* MOPH colony of the Department of Entomology (Faculty of Agriculture, Kasetsart University, Bangkok, Thailand) was used for laboratory tests where stage 3 larval reduction rates were calculated using different polymer disks or pellets in saucers.

For all tests, the mortality in the controls was very low, thus indicating that colonized mosquitoes used in this study were healthy at time of testing.

With the polymer disks in saucers in presence of 50 stage 3 larvae, we observed a strong mortality with disks impregnated with IKHC. This mortality was maximum with disks lot named “Juillet2010” impregnated with IKHC only, reaching 100 % from D3 onwards. The mortality decreases when IKHC is diluted with different synthetic geraniol. With pure synthetic geraniol (lots 1903/2 and /3), we observed a very low mortality. That means that the geraniol alone is probably not the active larvicide ingredient of IKHC. This test has been repeated after one and two months, using the same disks kept dry at room temperature. The larval reduction rate decreased dramatically from the first month, even with lot “Juillet2010” where the mortality dropped from 100 % to 19 % on day 6. That shows that the disks could not be used several times.

However, the duration of the effect during the first test (Table II) has not been evaluated beyond six days.

The efficiency of the IKHC is confirmed in experiment B using pellets. The mortality reached 100 % with 1 g of pellets in 500 ml of water at D3 (Table III) and this efficiency was conserved with pellets kept at room temperature in their original bag after one and two months (Tables III bis and III ter). The duration of the effect still needs to be evaluated to determine the frequency of use of pellets in saucers or different water collections in gardens.

The Experiment C was conducted in open field, in an area with wild *Ae. albopictus* populations, and it shows again a high efficiency of IKHC impregnated disks. In the control saucers, we observed new larvae at D6, D15 and D9 during the first, second and third tests respectively (Tables IV, IV bis and IV ter). In the saucers with IKHC impregnated disks, no larva was observed until the end of the test at D18 in all 3 tests for lot “Juillet2010” (Tables IV, IV bis and IV ter). There are two possibilities for this absence of larvae during the experiment; either the product is a repellent for females *Ae. albopictus* avoiding egg-laying in those saucers, or the product is also ovicide and all eggs have been killed before the emergence of larvae. These two hypotheses need now to be checked.

Finally, these polymer disks or pellets impregnated with natural products with insecticide activity could be an efficient tool to control mosquito populations in gardens, terraces or greenhouses. The pellets made of polymer and wood fibers seem especially practical for an individual usage. Our results showed that IKHC presents a very strong larvicide activity and, on the contrary, synthetic geraniol has no larvicide activity. As we do not know the active ingredient(s) of this product because the manufacturer refused to give us the complete and exact composition of the product, we therefore cannot recommend it for the moment. The way is opened to discover a natural product, probably a mixture of monoterpenes with larvicide activity which could be used with those slow release polymers or wood fiber systems. The advantage of using natural product against mosquito vectors is that they do not develop resistance and they are non-toxic to fishes, mammals and most other non-target organisms in the environment. As larvicide in mosquito control, insect growth regulator, biopesticides (Rozendaal, 1997), essential oil and natural product like mixture of monoterpenes (Ben Marzoug *et al.*, 2011; Guerrini *et al.*, 2011; Mkaddem *et al.*, 2009; Sacchetti *et al.*, 2004) represent alternative products for mosquito vector control.

## References

[R1] Ben Marzoug H.N., Romdhane M., Lebrihi A., Mathieu F., Couderc F., Abderraba M., Khouja M.L. & Bouajila J. *Eucalyptus oleosa* essential oils: chemical composition and antimicrobial and antioxidant activities of the oils from different plant parts (stems, leaves, flowers and fruits). Molecules, 2011, 16, 1695–1709 21330958 10.3390/molecules16021695 PMC6259913

[R2] Chuaycharoensuk T., Juntarajumnong W., Boonyuan W., Bangs M.J., Akratanakul P., Thummapalo S., Jirakanjanakit N., Tanasinchayakul S. & Chareonviriyaphap T. Frequency of pyrethroid resistance in *Aedes aegypti* and *Aedes albopictus* (Diptera: Culicidae) in Thailand. Journal of Vector Ecology, 2011, 36, 204–212 21635659 10.1111/j.1948-7134.2011.00158.x

[R3] Fontenille D., Lagneau C., Lecollinet S., Lefait-Robin R., Setbon M., Tirel B. & Yébakima A. La lutte antivectorielle en France – Disease control in France. Coll. Expertise collégiale, IRD Éditions, Paris, France, 2009, 533 p

[R4] George D.R., Biron J.M., Jolly G., Duvallet G. & Sparagano O.A.E. Toxicity of geraniol solution *in vitro* to the poultry red mite, *Dermanyssus gallinae*. Parasite, 2009, 16, 319–321 20092065 10.1051/parasite/2009164319

[R5] Guzman G.M., Halstead S.B., Artsob H., Buchy P., Farrar J., Gubler D.J., Hunsperger E., Kroeger A., Margolis H., Martínez E., Nathan M.B., Pelegrino J.L., Simmons C., Yoksan S. & Peeling R.W. Dengue: a continuing global threat. Nature Reviews Microbiology, 2010, 328, 745–748 10.1038/nrmicro2460 PMC4333201 21079655

[R6] Guerrini A., Rossi D., Paganetto G., Tognolini M., Muzzoli M., Romagnoli C., Antognoni F., Vertuani S., Medici A., Bruni A., Useli C., Tamburini E. & Sacchetti G. Chemical characterization (GC/MS and NMR Fingerprinting) and bioactivities of South-African *Pelargonium capitatum* (L.) L’Her. (Geraniaceae) essential oil. Chemistry & biodiversity, 2011, 8, 624–642 21480508 10.1002/cbdv.201000045

[R7] Khallaayoune K., Biron J.M., Chaoui A. & Duvallet G. Efficacy of 1 % geraniol (Fulltec^®^) as a tick repellent. Parasite, 2009, 16, 223–226 19839268 10.1051/parasite/2009163223

[R8] Kongmee M., Prabaripai A., Akratanakul P., Bangs M.J. & Chareonviriyaphap T. Behavioral responses of *Aedes aegypti* (Diptera: Culicidae) exposed to deltamethrin and possible implications for disease control. Journal of Medical Entomology, 2004, 41, 1055–1063 15605644 10.1603/0022-2585-41.6.1055

[R9] Mkaddem M., Bouajila J., Ennajar M., Lebrihi A., Mathieu F. & Romdhane M. Chemical composition and antimicrobial and antioxidant activities of *Mentha* (*longifolia* L. and *viridis*) essential oils. Journal of Food Science, 2009, 74, 358–363 10.1111/j.1750-3841.2009.01272.x 19895481

[R10] PPAV Working Group Personnal protection against biting insects and ticks. Parasite, 2011, 18, 93–111 21395212 10.1051/parasite/2011181093 PMC3671406

[R11] Rozendaal J.A. Vector control: methods for use by individuals and communities. World Health Organization, Geneva, Switzerland, 1997, 412 p

[R12] Sacchetti G., Medici A., Maietti S., Radice M., Muzzoli M., Manfredini S., Braccioli E. & Bruni R. Composition and functional properties of the essential oil of amazonian basil, *Ocimum micranthum* Willd., Labiatae in comparison with commercial essential oils. Journal of Agricultural and Food Chemistry, 2004, 52, 3486–3491 15161220 10.1021/jf035145e

